# Antibodies to Human Thymic Epithelium Form Part of the Murine Autoreactive Repertoire

**DOI:** 10.1155/2001/59678

**Published:** 2001

**Authors:** Jona Freysdottir, Mary A. Ritter

**Affiliations:** 1 Dept. of Immunology Imperial College School of Medicine Hammersmith Hospital Du Cane Road London W12 0NN UK; 2 Lyfjathroun hf, Biopharmaceutical Research Geirsgata 9 101 Reykjavik Iceland

**Keywords:** Autoantibodies, Epithelium, Microenvironment, Monoclonal Antibodies, Thymus

## Abstract

Monoclonal antibody (mAb) MR6 recognises a 200 kDa glycoprotein, gp200-MR6, which is expressed at high levels on the surface of human thymic cortical epithelium. In order to produce further mAbs against the gp200-MR6 molecule, mice were immunised with purified human gp200-MR6, hybridomas produced and supernatants screened for MR6-like reactivity on human thymic sections. Surprisingly this conventional hybridoma technique failed to produce stable hybridoma cells producing MR6-like antibodies. However, antibodies with specificitie other than MR6-like were obtained. Three such antibodies (1B2, 3A3 and 4B3) were analysed further. Expression of 1B2-antigen, 3A3-antigen and 4B3-antigen was analysed on skin, tonsil and thymic sections, on cultured thymic epithelial cells (TEC), thymocytes and peripheral blood mononuclear cells (PBMC), and found to be expressed by both lymphocytes and epithelial cell populations. Furthermore, the antigens were also expressed on mouse thymic, epithelial cells. The regulation of expression of these antigens was analysed following mitogen or cytokine stimulation of PBMC and cultured TEC, respectively. Expression on T cells was clearly affected by mitogens that mimic activation through the T cell receptor and expression on cultured TEC was affected by T cell-derived cytokines. Thus, the shared epithelial- lymphocyte molecules identified in this study may play a role in the cross-talk between the developing thymocytes and their epithelial microenvironment. The production of mAbs with specificities other than that of purified gp200-MR6 indicates that a wide range of B cells with specificity for components of the human thymic microenvironment exist in the normal mouse. These may detect epitopes that are shared with common pathogens to which the animals are exposed. Alternatively, they may be autoreactive B cells that are normally silent in the absence of T cell help. This help may be provided by T cells specific for human gp200-MR6, or nonspecifically by polyclonal activation induced by the adjuvant.


					Developmental Immunology, 2001, Vol. 8(2), pp. 75-93
Reprints available directly from the publisher
Photocopying permitted by license only
(C) 2001 OPA (Overseas Publishers Association)
N.V. Published by license under
the Harwood Academic Publishers imprint,
part of The Gordon and Breach Publishing Group,
member of the Taylor and Francis Group
Antibodies to Human Thymic Epithelium Form Part
of the Murine Autoreactive Repertoire
JONA FREYSDOTTIR* and MARY A. RITTERt
Dept. ofImmunology, Imperial College School ofMedicine, Hammersmith Hospital, Du Cane Road, London W12 ONN, England
Monoclonal antibody (mAb) MR6 recognises a 200 kDa glycoprotein, gp200-MR6, which is
expressed at high levels on the surface of human thymic cortical epithelium. In order to pro-
duce further mAbs against the gp200-MR6 molecule, mice were immunised with purified
human gp200-MR6, hybridomas produced and supernatants screened for MR6-1ike reactivity
on human thymic sections. Surprisingly this conventional hybridoma technique failed to pro-
duce stable hybridoma cells producing MR6-1ike antibodies. However, antibodies with spe-
cificitie other than MR6-1ike were obtained. Three such antibodies (1B2, 3A3 and 4B3) were
analysed further. Expression of 1B2-antigen, 3A3-antigen and 4B3-antigen was analysed on
skin, tonsil and thymic sections, on cultured thymic epithelial cells (TEC), thymocytes and
peripheral blood mononuclear cells (PBMC), and found to be expressed by both lymphocytes
and epithelial cell populations. Furthermore, the antigens were also expressed on mouse
thymic,epithelial cells. The regulation of expression of these antigens was analysed following
mitogen or cytokine stimulation of PBMC and cultured TEC, respectively. Expression on T
cells was clearly affected by mitogens that mimic activation through the T cell receptor and
expression on cultured TEC was affected by T cell-derived cytokines. Thus, the shared epi-
thelial-lymphocyte molecules identified in this study may play a role in the cross-talk
between the developing thymocytes and their epithelial microenvironment. The production of
mAbs with specificities other than that of purified gp200-MR6 indicates that a wide range of
B ceils with specificity for components of the human thymic microenvironment exist in the
normal mouse. These may detect epitopes that are shared with common pathogens to which
the animals are exposed. Alternatively, they may be autoreactive B cells that are normally
silent in the absence of T cell help. This help may be provided by T cells specific for human
gp200-MR6, or nonspecifically by polyclonal activation induced by the adjuvant.
Keywords: Autoantibodies, Epithelium, Microenvironment, Monoclonal Antibodies, Thymus
Abbreviations: Con A, concanavalin A, CTES, cluster of thymic epithelial staining, GlcNAc,
N-acetyl-l-D-glucosamine, mAb, monoclonal antibody, MFI, mean fluorescence intensity, PBMC,
peripheral blood mononuclear cells, PHA, phytohemagglutinin, PMA, phorbol 12-myristate 13-acetate,
PWM, pokeweed mitogen, TEC, thymic epithelial cells
* Present Address: Lyfjathroun hf, Biopharmaceutical Research, Geirsgata 9, 101 Reykjavik, Iceland.
f To whom correspondence should be addressed.
75
76 JONA FREYSDOTTIR and MARY A. RITTER
INTRODUCTION
The thymic microenvironment plays an important
role in thymocyte development by providing signals
regarding migration, differentiation, selection and
proliferation. These signals may be mediated by solu-
ble molecules produced by the stromal cells or by
direct cell-cell interaction between surface molecules
on the stromal cells and the thymocytes. However, little
is known about the molecular mechanism involved.
One way of investigating such microenvironmental
signals provided by the stromal cells is to analyse in
detail their surface molecules using monoclonal anti-
body and phage display techniques (van Vliet et al.,
1984; de Maagd et al., 1985; Boyd et al., 1993;
Palmer et al., 1997). The reagents generated in these
studies have revealed significant molecular heteroge-
neity within the epithelial component of the thymic
microenvironment.
Antibodies raised against the thymic epithelium of a
variety of species, including human, have been divided
into five major clusters of thymic epithelial staining
(CTES) patterns (Kampin,ga et al., 1989; Ritter and
Crispe, 1992). CTES I antibodies are pan-epithelial,
CTES II antibodies label subcapsular/perivascular and
a major subpopulation of medullary epithelial cells,
CTES III antibodies detect molecules on cortical epi-
thelium, CTES IV antibodies label Hassall's corpus-
cles and all medullary epithelial cells, and CTES V
reagents stain Hassall's corpuscles and sometimes a
surrounding halo of medullary epithelial cells. How-
ever, for only few of these mAbs are their target anti-
gens identified.
One such antibody is the mAb MR6. The molecule
recognised by mAb MR6, gp200-MR6, is a 200 kDa
glycoprotein expressed at high levels on the surface
of human cortical epithelium and at much lower lev-
els on macrophages, dendritic cells and thymocytes
(Larch6 et al., 1988a; Mat et al., 1990). Apart from
the thymus the gp200-MR6 is expressed on peripheral
lymphocytes and on epithelial cells in breast, colon
and skin (Larch6 et al., 1988a; A1-Tubuly et al.,
1996). Since gp200-MR6 is shared by thymocytes
and epithelial cells it was a potential candidate as a
molecule participating in cell-cell cross-talk between
the thymocytes and the epithelium. Functional analy-
sis revealed that addition of mAb MR6 to cultures of
T cell clones partially inhibited both antigen- and
IL-2-induced proliferation and completely blocked
IL-4-induced proliferation in a dose-dependent man-
ner and that mAb MR6 completely abrogated the
IL-4-dependent production of specific anti-
gen-induced IgE by B cells (Larch6 et al., 1988a,
1988b). This dramatic influence on IL-4-dependent
functions suggested that the gp200-MR6 was linked
to the IL-4 receptor. Furthermore, mAb MR6 has
been found to inhibit alloreactivity, probably via a
block in the expansion of IL-4 responsive/secreting
Th2 helper T cells (Imami et al., 1994). However,
recent studies suggest that although mAb MR6 may
antagonise the proliferation effect of IL-4 on lym-
phocytes it appears to behave as an agonist for the dif-
ferentiation effect of IL-4 on epithelial cells
(A1-Tubuly et al., 1997).
These studies raised the interesting possibility that
the gp200-MR6 molecule may be involved in differ-
ential signalling via the IL-4 receptor, and that its
high expression on cortical epithelium may reflect an
important role in maintenance/differentiation of the
cortical thymic microenvironment via thymo-
cyte-derived IL-4. However, since the gp200-MR6
contains 180 kDa of protein it is likely that the mole-
cule contains more than one functional domain. Fur-
ther studies would, therefore, be greatly facilitated by
the development of mAbs to different regions of the
gp200-MR6. Therefore, in order to be able to study
the structure and function of the molecule further and
to elucidate its link with the IL-4 receptor, mice were
immunised with purified gp200-MR6 and their splen-
ocytes used to produce hybridoma cells producing
new monoclonal antibodies against gp200-MR6. Sur-
prisingly, although no anti-gp200-MR6 secreting cells
survived, several hybridoma cell lines secreting anti-
bodies with specificities other than those that were
MR6-1ike were obtained indicating that antibodies to
thymic epithelium may form part of the murine auto-
reactive repertoire.
ANTIBODIES TO HUMAN THYMIC EPITHELIUM 77
78 JONA FREYSDOTTIR and MARY A. RITTER
RESULTS
Production of new Monoclonal Antibodies against
Gp200-MR6
Immunisation with purified human gp200-MR6
resulted in highly immunised mice, as confirmed by
testing sera or ascites obtained from these mice. Sub-
sequently, hybridoma cells secreting MR6-1ike anti-
bodies were produced. This was confirmed by testing
the hybridoma supernatants on thymic tissue sections.
Unfortunately, these hybridoma cells always died,
usually during the first cloning procedure, when cells
had started to proliferate in the small wells in the 96
well plates.
Production of new Monoclonal Antibodies
with Different Specificities than MR6-1ike
Screening the hybridoma cell supernatants on human
thymic sections allowed detection of antibodies with
specificities other than those that were MR6-1ike.
Interestingly, hybridomas secreting antibodies with
various specificities were detected. Amongst these
were antibodies staining the thymocytes, different
populations of thymic epithelium, thymocytes and
epithelium, macrophages or dendritic cells, blood
vessels and soluble particles around them, and finally
antibodies that stained all cells as well as the intercel-
lular areas.
Three hybridomas which stained different subpop-
ulations of thymic epithelium were selected for fur-
ther studies, 1B2, 3A3 and 4B3. These hybridomas
were cloned at least three times, the last time with 0.3
cells per well. When single clones were obtained
these were grown in bulk and a large amount of super-
natant collected.
Distribution of the Molecules Recognised
by the new Monoclonal Antibodies
Human thymic, skin and tonsil sections, PBMC, thy-
mocytes, and cultured thymic epithelial cells were
stained with the new monoclonals, in order to analyse
the distribution of the molecules they recognise
within lymphoid and non-lymphoid organs (table I).
For comparison, mouse thymic epithelial cells were
also stained with the new monoclonals.
Expression within the thymus
The expression of the molecules that were recognised
by the new antibodies was studied on thymic sections
(table I, figure 1). MAb 1B2 stained only some med-
ullary epithelial cells. MAb 3A3 stained subcapsular,
medullary and some cortical epithelial cells, as well
as Hassall's corpuscles; it also stained thymocytes
very weakly. MAb 4B3 stained Hassall's corpuscles
very strongly and also thymocytes. No staining on
thymocytes or epithelium was observed with the iso-
type matched antibody mAb 8D4 (data not shown).
Expression within other tissues than thymus
All three monoclonal antibodies stained tonsil and
skin sections (table I, figure 2). MAb 1B2 stained the
basal layer of the epidermis (basal epithelium) and the
basal cell layer of the epithelial folds within the tonsil
(figures 2A and 2B). MAb 4B3, on the other hand,
stained mostly the upper layer of the epidermis (squa-
mous epithelium) and the outer cell layer of the epi-
thelial folds within the tonsil (figures 2E and 2F).
MAb 3A3 stained more or less all the epithelial cells
in the skin and tonsil (figures 2C and 2D), with a sim-
ilar staining distribution as that observed for cytok-
eratins (figures 21 and 2J). No staining of the
epithelium was observed with the isotype matched
control (mAb 8D4) although blood vessels in the ton-
sils were positive (figures 2G and 2H). MAbs 1B2,
3A3 and 4B3 all stained the cytoplasm, although an
apparent surface staining was observed for mAb 3A3
on squamous epithelium in the skin.
Expression on thymocytes and PBMC
Expression of the molecules recognised by the new
monoclonal antibodies was analysed on/in PBMC
from three healthy individuals and on thymocytes
from two paediatric thymuses. Expression of all the
antigens was found to be intracellular, both when cells
were analysed by immunofluorescence and flow
80 JONA FREYSDOTTIR and MARY A. RITTER
gen varied between individuals, with 10 to 50% of
PBMC and thymocytes being positive when analysed
by immunofluorescence, although cytospin analysis
indicated that essentially all cells contained the
1B2-antigen. All PBMC expressed the 3A3-antigen
and 4B3-antigen when analysed on cytospins and
approximately 80% of PBMC and thymocytes were
positive for 3A3-antigen and 4B3-antigen when ana-
lysed by immunofluorescence. The staining intensity
for the 3A3-antigen was very high, more than ten
times stronger than that for the 4B3-antigen, both
observed by immunofluorescence and on cytospins.
The patterns obtained by immunoenzyme staining
of PBMC with mAbs 1B2, 3A3 and 4B3 were quite
different from each other. MAb B2 stained the cyto-
plasm homogeneously (figure 4A). MAb 3A3, on the
other hand, stained strongly patches in the cytoplasm
located next to the nucleus, as well as the area next to
the nuclear membrane (figure 4B). MAb 4B3 had a
granular staining pattern with some of the granules
accumulated next to the nuclear membrane
(figure 4C). Double immunoenzyme labelling
revealed that both B and T lymphocytes expressed the
1B2-antigen, 3A3-antigen and 4B3-antigen (data not
shown). No cell staining of PBMC was observed with
the isotype matched control although the platelets
were positive with this mAb (figure 4D).
Expression on cultured thymic epithelial cells
Primary cultures of human thymic epithelial cells,
obtained from tissue explants, grown in slide flasks,
were analysed for expression of the molecules recog-
nised by the new monoclonal antibodies (table I,
figure 5). All cells expressed the 3A3-antigen and
4B3-antigen, whereas the majority of the cells
expressed the 1B2-antigen. As can be seen in figure 5
the expression was predominantly internal, although
the staining pattern did vary for the different mAbs.
MAbs 1B2 and 3A3 both stained all the cytoplasm,
where lB2-antigen was expressed weakly, but quite
homogeneously within the cells (figure 5A) compared
to 3A3-antigen which was expressed strongly in
patches (figure 5B). Expression of 4B3-antigen
clearly had a granular pattern with the granules accu-
mulating next to the nucleus (figure 5C). No staining
was observed with the isotype matched antibody mAb
8D4 (figure 5D). For comparison, mouse thymic epi-
thelial cells cultured in slide flasks were also stained
with the new monoclonal antibodies (figure 6). Inter-
estingly, the same staining pattern was obtained as
with the human thymic epithelial cells.
Regulation of Expression of the Molecules
Recognised by the new Monoclonal Antibodies
By mitogens on PBMC
PBMC from three healthy individuals were cultured
in the presence of medium alone, PHA, Con A, PWM
or PMA plus ionomycin. The cells were collected at
different time points (4, 24, 48 and 72 hours), fixed
and stained in the presence of 0.1% saponin with
mAbs 1B2, 3A3 and 4B3 (figure 7) and with antibod-
ies against CD3, CD20, CD23 and CD25 for compar-
ison (data not shown).
Only PHA upregulated expression of CD3,
whereas PHA, PWM and PMA plus ionomycin
caused an upregulation in CD23 expression. All the
mitogens used caused an upregulation in CD25
expression.
The percentage of PBMC expressing 1B2-antigen
declined with increased time in culture (from 32 to
8%) and this decline was not affected by Con A or
PWM. On the other hand, PHA and PMA plus iono-
mycin caused an increase in percentage' of positive
cells (from 32 to 53% for PHA and from 32 to 45%
for PMA plus ionomycin). However, this was only
observed after 48 hours, with values already back to
baseline levels after 72 hours.
Expression of 3A3-antigen and 4B3-antigen
decreased even more rapidly with increased time in
culture than that observed for B2-antigen (from 86
to 41% and 84 to 26% for 3A3-antigen and 4B3-anti-
gen, respectively). This decline in the percentage of
positive wells was, though, not observed when cells
were cultured in the presence of PHA.
82 JONA FREYSDOTTIR and MARY A. RITTER
TABLE II Influence of cytokines on expression of 1B2-antigen, 3A3-antigen and 4B3-antigen on cultured thymic epithelial cells
Medium IL-2 IL-4 IL-13 IFN-y
1B2-antigen +/- no change
" '" no change
3A3-antigen ++ no change
" no change
"
4B3-antigen ++
'" no change no change
"
negative; +/-, some cells positive; +, all cells positive with medium staining intensity; ++, all cells positive with strong staining intensity;
', upregulation of expression; '', strong upregulation of expression.
By cytokines on cultured thymic epithelial cells
Thymic epithelial cells were cultured in slide flasks in
the presence of medium alone, IL-2, IL-4, IL-13 or
IFN-y. Then the cells were stained with mAbs 1B2,
3A3 and 4B3 and with mAb 8D4 as an isotype spe-
cific control, and the expression of the 1B2-, 3A3-
and 4B3-antigens analysed by immunoperoxidase
staining (table II, figure 8). IL-2 and, to a lesser
extent, IFN-y caused an increase in staining intensity
of the 4B3-antigen, whereas IL-13 and, to a lesser
degree, IL-4 caused an increase in staining intensity
of the 1B2-antigen. Furthermore, IFN-y and IL-4
caused an increase in staining intensity of 3A3-anti-
gen.
DISCUSSION
Immunisation with purified gp200-MR6 was very
successful. Already 10 days after the second immuni-
sation, the sera from the mice contained large quanti-
fies of MR6-1ike antibodies (effective in
immunohistochemical staining at a dilution of
1/4000). In order to allow class switching and affinity
maturation to occur, the mice were immunised twice
more, at intervals of at least 2 weeks.
All fusions resulted in production of hybridoma
cells producing MR6-1ike antibodies. Unfortunately,
these hybridoma cells always died, usually at the first
cloning stage when cells started to proliferate in the
small wells in the 96 well plates. It can be suggested
that in the small wells the antibodies were present at
high concentration and somehow toxic for the cells.
Since B cells use IL-4 as a growth factor it is possible
that antibodies which cross-react with the murine
gp200-MR6 molecule (i.e. autoreactive) were block-
ing cell growth. Thus, it seem likely that conventional
hybridoma technology is unlikely to yield more
MR6-1ike antibodies. This has, however, been suc-
cessful using phage-display technology (Palmer et al.,
1999).
Surprisingly, each fusion produced hybridoma cells
secreting antibodies with specificities other than those
that were MR6-1ike. Amongst the specificities
detected were staining of all or some epithelial cells,
of thymocytes, of thymocytes and some/all epithelial
cells, of blood vessels, of macrophages and of inter-
cellular tissue. This diversity in staining pattern was
very surprising, since purified gp200-MR6 had been
used for immunisation. One explanation could be that
the gp200-MR6 was indeed not completely pure. This
is unlikely, since eluates run on SDS/PAGE and
stained with Coomassie blue did not reveal any addi-
tional bands other than those recognised by mAb
MR6. Another explanation is that the polyclonal acti-
vation, caused by the adjuvant, stimulated autoreac-
tive B cells, producing antibodies that cross-reacted
with human molecules. During evolution, many
important proteins have conserved genes that can be
found in unrelated species. Rabbit antibodies against
human cytokeratins or laminins can, for example, be
used to stain mouse cytokeratins or laminins (per-
sonal observation). Previous studies, confirming this
suggestion, have shown that supernatants of hybrido-
mas, obtained from normal, germ free, nude (ath-
ymic) and neonatal mice, produced antibodies
reactive with intracellular structures in mouse, rat and
human fibroblasts or autologous macromolecules,
such as actin, tubulin, myosin and dsDNA) (Under-
wood et al., 1985; Lymberi et al., 1989). Interestingly,
these auto-reactive hybridomas could be obtained
from both primed and unprimed mice.
ANTIBODIES TO HUMAN THYMIC EPITHELIUM 83
100
60-
40-
2O
0
100-
80-
60-
40-
20-
AA
HL
JF
HT50
% pos cells MFI
% pos cells MFI*
100
80-
60
4O
20
% pos cells MFI
100
80 HL
60
JF
HTS0
40- HT51
20-
% pos cells MFI
1B2
3A3
4B3
8D4
FIGURE 3 Intracellular expression of molecules recognised by mAbs 1B2, 3A3 and 4B3 and an isotype matched control (mAb 8D4) on
PBMC (AA, HL, JF) and thymocytes (HT50, HT51), analysed by immunofluorescence and flow cytometry on fixed and saponin treated
cells, with results expressed as percentage positive cells or mean fluorescence intensity (MFI). * indicates that actual MFI is 10 times higher
than levels shown
Molecular mimicry could also be an explanation
for the occurrence of the non MR6-1ike antibodies.
This has been observed for antibodies against the
streptococcal A carbohydrate, terminal O-linked
N-acetyl--D-glucosamine (GlcNAc), produced dur-
ing streptococcal infection, which cross-react with
ANTIBODIES TO HUMAN THYMIC EPITHELIUM 85
Furthermore, mouse anti-streptococcal antibodies
expressing anti-GlcNAc activity were found to react
with several cytoskeletal proteins, such as actin,
vimentin, myosin and cytokeratin (Shikman et al.,
1993). Further studies with monoclonal antibodies
against GlcNAc or to cytokeratin (Shikman and Cun-
ningham, 1994, Shikman et al., 1994) suggested that
certain peptides, containing O-linked GlcNAc, could
induce an anti-carbohydrate antibody response in
vivo. All the new monoclonal antibodies were IgM,
suggesting that they might be against carbohydrates.
An internal infection amongst the mice could provoke
such an immune response. On the other hand,
although gp200-MR6 only contains N-linked sugars it
could act as the non-cytokeratin molecules containing
O-linked GlcNAc described by Shikman and Cun-
ningham (1994) which induced antibodies against
cytokeratin in BALB/c mice.
Three hybridomas secreting non MR6-1ike antibod-
ies were selected for further studies. MAbs 1B2, 3A3
and 4B3 stained epithelial cells in human thymus,
tonsil and skin. MAb 3A3 stained all epithelial cells
in thymus, epidermis and tonsil, a staining pattern
also observed for cytokeratins (personal observa-
tions). MAb 1B2 stained only some medullary epithe-
lial cells in the thymus and the basal layer of the
epidermis of the skin and the basal layer of the epithe-
lial folds of the tonsils. MAb 4B3, on the other hand,
stained Hassall's corpuscles in the thymus and the
upper layer of the epidermis (squamous epithelium)
and the outer cell layer of the epithelial folds within
the tonsil. Many monoclonal antibodies against Has-
sail's corpuscles have been produced using crude thy-
mus or peptides based on the gene sequence of the
IL-4 receptor as immunogens and the majority of
these antibodies were IgM and likely to be against
carbohydrates (de Maagd et al., 1985; Mat, 1992;
Imami et al., 1997). However, an antibody recognis-
ing only some medullary thymic epithelial cells adds
further proof for the existence of subpopulations of
medullary epithelial cells (van der Wijngaert et al.,
1984; Farr et al., 1993). The role of the medullary epi-
thelium in thymocyte development is still unknown. It
has been shown that some medullary thymocytes go
through cell division before leaving the thymus (Ernst
et al., 1995), suggesting that the medullary stromal
cells provide the necessary proliferative signals.
These signals might be provided by some of the med-
ullary epithelial cells. Furthermore, it has been shown
that medullary epithelial cells are able to induce nega-
tive selection of thymocytes (Volkmann et al., 1997).
By staining tissue sections it was observed that
mAbs 3A3 and 4B3 also stained thymocytes and lym-
phocytes, as seen on thymus and tonsil for mAb 4B3
and on thymus for mAb 3A3. This observation was
confirmed by staining thymocytes and PBMC in sus-
pension. Surprisingly, mAb 1B2 was also found to
stain thymocytes and PBMC, where 10-47% of the
cells from different individuals were positive when
analysed by immunofluorescence staining and flow
cytometry. Expression of 1B2-antigen was found to
be influenced by cytokines. Therefore, the variation
of percentage of 1B2-antigen positive cells from dif-
ferent individuals may reflect different cytokine regu-
lation. Double labelling studies revealed that all these
antibodies stained both B and T cells. This observa-
tion adds 1B2-antigen, 3A3-antigen and 4B3-antigen
to the group of molecules which are expressed on
both lymphoid and non-lymphoid cells and makes
them possible candidates participating in the cell-cell
cross-talk occurring in the thymus, as suggested by
Ritter and Boyd (1993). Several such molecules (e.g.
gp200-MR6 and MTS 12, 32, 33, 35 and 37) have
been investigated in further details (Larch6 et al.,
1988a; Tucek et al., 1992; A1-Tubuly et al., 1996).
All three molecules recognised by mAbs 1B2, 3A3
and 4B3 are expressed predominantly internally.
However, mAb 3A3 stained the surface of the squa-
mous epithelium in the skin, as well as the cytoplasm.
These are the most differentiated epithelial cells in the
skin, suggesting that 3A3-antigen is transported out
on the surface when the epithelial cells reach a more
mature stage. The staining patterns on PBMC and cul-
tured epithelial cells obtained by the different mAbs
varied considerably. MAb 1B2 stained the cytoplasm
quite homogeneously, mAb 3A3 stained patches in
the cytoplasm very strongly as well as the area
around/at the nuclear membrane and mAb 4B3
stained granules within the cytoplasm. The molecules
may be part of the intracellular structure; however, the
88 JONA FREYSDOTTIR and MARY A. RITTER
60
40
20
20 40
Hours
60 80
1B2-ag
Medium
PHA
-----0---- Con A
PWM
PMA+ionornycin
80
60
40
3A3-ag
Medium
PHA
Con A
PWM
PMA+ionomycin
20 40
Hours
60 80
80
60
40
20
4B3-ag
Medium
PHA
Con A
PMA+ionomycin
0 20 40 60 80
Hours
FIGURE 7 Influence of mitogens on expression of 1B2-antigen, 3A3-antigen and 4B3-antigen in/on PBMC from three individuals, analysed
after fixation by immunofluorescence and flow cytometry, with results expressed as percentage positive cells (mean standard deviation)
3A3-antigen and 4B3-antigen are all expressed on
lymphocytes and thymic epithelium, we analysed
whether mitogens or cytokines would affect their
expression on PBMC or cultured thymic epithelial
cells, respectively.
The expression of all the three antigens recognised
by mAbs 1B2, 3A3 and 4B3 in/on PBMC declined
with increased time in control cultures. The polyclo-
nal T cell activator, PHA, was the only mitogenic
compound that could inhibit the decline in expression
ANTIBODIES TO HUMAN THYMIC EPITHELIUM 89
of 3A3-antigen and 4B3-antigen. On the other hand,
PHA and PMA plus ionomycin and, to a lesser extent,
Con A and PWM caused an increase in expression of
1B2-antigen, observed only after 48 hours in culture.
When the cells were analysed 24 hours later this
increase was barely detectable. PHA and Con A only
activate T cells, most likely by binding to component
chains of the TCR/CD3 complex (Kanellopoulos et
al., 1985). Therefore, expression of 1B2-antigen,
3A3-antigen and 4B3-antigen on T cells must be
affected by the activation stage. PMA plus ionomycin
can mimic some of the events that are associated with
TCR stimulation (Truneh et al., 1985) and cross-link-
ing of membrane bound Ig (Gold et al., 1991) and
PWM does also activate both T and B cells. There-
fore, it cannot be concluded whether mitogens ..also
affect expression of 1B2-antigen on B cells as well as
on T cells.
All cultured human thymic epithelial cells express
3A3-antigen and 4B3-antigen and the majority of the
cells express 1B2-antigen but very weakly. Increased
staining intensity was observed for 1B2-antigen if the
cells were treated with IL-4 or IL-13. It has been pro-
posed that IL-4 and IL-13 induce maturation of cul-
tured thymic epithelial cells (Freysdottir and Ritter,
manuscript in preparation). The findings reported
here would, therefore, suggest that expression of the
molecules recognised by mAb 1B2 is influenced by
the maturation stage of the epithelial cells, with
upregulation in expression with increased maturation.
In contrast, expression of the 4B3-antigen was
increased when cells were cultured in the presence of
IL-2 or IFN-,, while treatment with either IL-4 or
IFN-/ caused a slight upregulation of 3A3-antigen
expression, showing that both Thl and Th2 cytokines
could influence the 3A3-antigen expression. These
results indicate that expression of the three molecules
recognised by mAbs 1B2, 3A3 and 4B3 is differen-
tially regulated by cytokines produced within the thy-
mus, raising the possibility that thymocyte
subpopulations might control development of distinct
microenvironmental nichies.
In summary, we have identified three novel mole-
cules that are expressed in human thymus by both
developing thymocytes and their epithelial microen-
vironment. This shared distribution and the regulation
of their expression by T cell-derived cytokines sug-
gest that these molecules may be important in intrath-
ymic cross-talk. Future studies will focus on their
structure and function.
MATERIALS AND METHODS
Purification of Gp200-MR6
Gp200-MR6 was purified from lysates of human thy-
mus by affinity chromotography using an Immunopure(R)
Ag/Ab Immobilization Kit (Pierce & Warriner, Ches-
.ter, England). The mAb MR6 (4.6 mg/ml in 50 mM
sodium acetate buffer, pH 5.5) was immobilised to the
agarose beads according to the manufacturer's proto-
col. The MR6-agarose was finally divided into 500 tl
aliquots.
Frozen sections of human thymic tissue (obtained
from children aged 5 days to 8 years that were under-
going cardiac surgery at the Great Ormond Street
Hospital for Sick Children and Royal Brompton Hos-
pital, London) were solubilised and used as the source
of gp200-MR6, as previously described by A1-Tubuly
et al. (1996). Approximately 100 sections, 18 ktm
thick, were cut in a cryostat, placed into an ice cold
tube and then solubilised in 1.2 ml of lysis buffer (10
mM Tris-HC1, pH 7.2, containing 1% Nonidet P-40
and 1 mM phenylmethyl-sulfonyl fluoride) for 15
minutes on ice. The tubes were centrifuged at 10,000
rpm at 4C for 15 minutes. The supernatant, contain-
ing the solubilised gp200-MR6, was collected and
either used immediately or stored at -20C.
The purification of gp200-MR6 was performed at
4C. 1 ml of thymic lysate was added into 500 tl of
MR6-agarose. The agarose was resuspended and the
tubes placed on a rotator for 40 minutes. The tubes
were then spun in a microfuge for several seconds and
the thymic lysate removed carefully. This was
repeated four times with flesh thymic lysate each
time. Then the agarose was washed six times with
1 ml PBS for 5 minutes. Elufion of gp200-MR6 was
performed by mixing 500 tl of 0.1 M glycine buffer,
ANTIBODIES TO HUMAN THYMIC EPITHELIUM 91
M Tris-HC1, pH 9.4, in order to adjust the pH to
approximately 7.5.
The presence of gp200-MR6 in the eluates was
confirmed by Western blotting. Membranes were
stained with mAb MR6 or second antibody alone
which should detect any contamination of mAb MR6
in the eluates. The amount of gp200-MR6 in the elu-
ates was estimated by measuring protein concentra-
tion by a microtiter plate version of the Lowry
method.
Production of Hybridoma Cells
BALB/c female mice between 6 and 8 weeks old
were immunised ip with 200 ktl of purified human
gp200-MR6 (approximately 20 tg) mixed with 100
ktl of complete Freund's adjuvant. The mice were
boosted twice at three to five week intervals with
purified gp200-MR6 mixed with incomplete Freund's
adjuvant. The final injection was with the purified
gp200-MR6 alone, four days before a fusion was per-
formed. During fusion the myeloma cells, NSO, were
added to the splenocytes at a ratio of one NSO mye-
loma cell per 6 to 10' splenocytes (a yield of 1.5 to 2.0
x 108 splenocytes was usually obtained). After the
fusion the cells were plated out into 24 well plates at a
concentration of 1 to 2 x 105 cells per well. Superna-
tants from wells that contained hybridoma cell clones
were screened for presence of MR6-1ike antibodies by
immunoperoxidase staining of thymic sections. Posi-
tive hybridoma cells were subsequently cloned three
times by limiting dilutions to ensure single clones.
Antibodies were isotyped and all shown to be IgM
(Isotyping kit, Serotec, Oxford, England).
Analysis of the Molecules Recognised by the new
Monodonal Antibodies
Sections prepared from human thymus, tonsils and
skin, cultured human TEC obtained from explants of
human thymic tissues (Freysdottir and Ritter, manu-
script in preparation), cultured mouse TEC (F1), and
cytospins of human PBMC were immunoperoxidase
stained with the hybridoma supernatants. For compar-
ison and positive control, the sections and cultured
human TEC were also stained with antibody against
cytokeratin 19 (clone K4.62; Sigma, Poole, England)
and an isotype matched control antibody (mAb 8D4,
labels endothelium and platelets only was added as
negative control). The cytospins were double stained
by the immunoperoxidase technique with hybridoma
supernatants and then by the alkaline phosphatase
technique with mAbs (all from Dako, High
Wycombe, England) against CD3 (clone T3-4B5) or
against CD19 (clone HD37), CD20 (clone B-Lyl) and
CD22 (clone 4KB128). Secondary antibodies were
rabbit-anti mouse Ig conjugated to either peroxidase
or alkaline phosphatase (Dako).
Cell suspensions of human PBMC and thymocytes
were stained by indirect immunofluoroscence with
hybridoma supernatants. MAb 8D4 was included as
an isotype matched antibody control. Rabbit
anti-mouse Ig labelled with fluorescein isothiocy-
anate (Dako) was used as secondary antibody. After
staining the cells were fixed in 1% paraformaldehyde
and analysed by flow cytometry (Epics XL, Coulter,
Hileah, USA). In order to analyse intracellular mole-
cules, cells were fixed with 1% paraformaldehyde for
10 minutes on ice prior to staining and 0.1% saponin
was included in all washing and incubation steps.
Results were expressed as percentage positive cells,
compared to cells stained with an isotype matched
control, or mean fluorescence intensity (MFI).
Regulation of the Expression of the Molecules
Recognised by the new Monoclonal Antibodies
Freshly isolated PBMC from three healthy individu-
als were incubated at 2 x 106 cells per well in a 24
well plate. The cells were cultured in medium alone
(RPMI medium supplemented with 10% foetal calf
serum) or in the presence of mitogens (Sigma), phyto-
hemagglutinin (PHA), used at 2 ktg/ml, concanavalin
A (Con A), used at 25 tg/ml, pokeweed mitogen
(PWM), used at 2.5 ktg/ml, and phorbol 12-myristate
13-acetate (PMA), used at 10 ng/ml together with 1
tg/ml of the calcium ionophore ionomycin. The con-
centrations used were all within the range recom-
mended by the manufacturer. Cells were harvested
92 JONA FREYSDOTTIR and MARY A. RITTER
after 4, 24, 48 and 72 hours and analysed by immun-
ofluorescenc and flow cytometry. Approximately 2 x
105 cells were incubated with mAbs (Dako) against
CD3, CD23 (clone MHM6) or CD25 (clone ACT-l)
for analysing surface expression or with hybridoma
supernatants for internal and/or surface expression.
Thymic epithelial cells, growing in slide flasks,
were cultured for 48 hours in the presence or absence
of IL-2 (20 U/ml, Boehringer Mannheim, Lewes,
England), IL-4 (50 U/ml, Genzyme Diagnostics,
Kings Hill, England), IL-13 (15 ng/ml, Pepro Tech,
Rocky Hill, NJ, USA) or IFN-/ (100 U/ml, Gen-
zyme). The cells were then stained by the indirect
immunoperoxidase technique using hybridoma super-
natants and analysed using light microscopy by two
independent observers. The intensity of staining of
untreated and treated cells was compared. Results
were given as no change (no change in staining inten-
sity), upregulation (increase in staining intensity) or
downregulation (decrease in staining intensity).
Acknowledgements
This work was supported by NATO Fellowship, Ida
MacLean Fellowship (International Federation of
University Women), Helga Jonsdottir and Sigurlidi
Kristjansson's Memorial Fund, Bergthora Magnusdot-
tir and Jakob Bjarnason's Memorial Fund, Theodor
Johnson's Memorial Fund, and Islandsbanki's Stu-
dentline.
References
A1-Tubuly, A.A., Luqmani, Y.A., Shousha, S., Melcher, D. and Rit-
ter, M.A. (1996) Downregulation of the IL-4 receptor-associ-
ated molecule gp200-MR6 in invasive carcinoma and
differential expression in benign hyperplasia of the breast. Br.
J. Cancer 74: 1005-1011.
A1-Tubuly, A.A., Spijker, R., Pignatelli, M., Kirkland, S. and Ritter,
M.A. Inhibition of proliferation and enhancement of differen-
tiation of human colorectal cell lines by MAb MR6 and inter-
leukin-4. Int. J. Cancer 71:605-611.
Boyd, R.L., Tucek, C.L., Godfrey, D.I., Izon, D.J., Wilson, T.J.,
Davidson, N.J., Bean, A.G.D., Ladyman, H.M., Ritter, M.A.
and Hugo, P. (1993) The thymic microenvironment. Immunol.
Today 14: 445-459.
de Maagd, R.A., Mackenzie, W.A., Schuurman, H.-J., Ritter, M.A.,
Price, K.M., Broekhuizen, R. and Kater, L. (1985) The human
thymus microenvironment: heterogeneity detected by mono-
clonal anti-epithelial cell antibodies. Immunology 54: 745-
754.
de Rose, V., Rolla, G., Bucca, C., Ghio, P., Bertoletti, M., Baderna,
P. and Pozzi, E. (1994) Intercellular adhesion molecule-1 is
upregulated on peripheral blood T lymphocyte subsets in dual
asthmatic responders. J. Clin. Invest. 94:1840-1845.
Ernst, B., Surh, C.D. and Sprent, J. (1995) Thymic selection and
cell division. J. Exp. Med. 182, 961-972.
Farr, A., Nelson, A., Hosier, S. and Kim, A. (1993) A novel
cytokine-responsive cells surface glycoprotein defines a sub-
set of medullary thymic epithelium in situ. J. Immunol. 150,
1160-1171.
Gold, M.R., Matsuuchi, L., Kelly, R.B. and DeFranco, A.L. (1991)
Tyrosine phosphorylation of components of the B-cell antigen
receptors following receptor crosslinking. Proc. Natl. Acad.
Sci. USA 88: 3436-3440.
Imami, N., Ladyman, H.M., Vincents, B., A1-Tubuly, A., Freysdot-
tir, J., Sedibane, M.L., Taylor-Fishwick, D.A., Foxwell, B. and
Ritter, M.A. (1998) K21-antigen a molecule shared by the
microenvironments of the human thymus and germinal cen-
tres. Devel. Immunol. 6:41-52.
Imami, N., Larchr, M. and Ritter, M.A. (1994) Inhibition of allore-
activity by mAb MR6: differential effects on IL-2-and
IL-4-producing human T cells. Int. Immunol. 6: 1575-1584.
Kampinga, J., Berges, S., Boyd, R.L., Brekelmans, P., Colic, M.,
van Ewijk, W., Kendall, M.D., Ladyman, H., Nieuwenhuis, P.,
Ritter, M.A., Schuurman, H.-J. and Tournefier, A. (1989)
Thymic epithelial antibodies: Immunohistological analysis
and introduction of nomenclature. Thymus 13: 165-173.
Kanellopoulos, J.M., De Petris, S., Leca, G. and Crumpton, M.J.
(1985) The mitogenic lectin from Phaseolus vulgaris does not
recognize the T3 antigen of human T lymphocytes. Eur. J.
Immunol. 15: 479-486.
Larchr, M., Lamb, J.R. and Ritter, M.A. (1988a) A novel T-lym-
phocyte molecule that may function in the induction of
self-tolerance and MHC-restriction within the human thymic
microenvironment. Immunology 64: 101-105.
Larchr, M., Lamb, J.R., O'Hehir, R.E., Imami-Shita, N., Zanders,
E.D., Quint, D.E., Moqbel, R. and Ritter, M.A. (1988b) Func-
tional evidence for a monoclonal antibody that binds to the
human IL-4 receptor. Immunology 65:617-622.
Lymberi, P., Blancher, A., Calvas, P. and Avrameas, S. (1989) Nat-
ural autoantibodies in nude and normal outbred (Swiss) and
inbred mice (BALB/c) mice. J. Autoimmunity 2: 283-295.
Marker, O., Scheynius, A., Christensen, J.P. and Thomsen, A.R.
(1995) Virus-activated T cells regulate expression of adhesion
molecules on endothelial cells in sites of infection. J. Neu-
roimmunol. 62: 35-42.
Mat, I., Larchr, M., Melcher, D. and Ritter, M.A. (1990)
Tumour-associated upregulation of the IL-4 receptor complex.
Br. J. Cancer (suppl) 10: 96-98.
Mat, I. (1992) Analysis of human interleukin 4 receptor-associated
molecule (gp200-MR6 molecule) in normal and transformed
epithelium. PhD thesis. University of London.
Palmer, D.B., George, A.J.T. and Ritter, M.A. (1997) Selection of
antibodies to cell surface determinants on mouse thymic epi-
thelial cells using a phage display library. Immunology 91:
473-478.
Palmer, D.B., Crompton, T., Marandi, M.B., George, A.J.T. and
Ritter, M.A. (1999) Intrathymic function of the human cortical
epithelial cell surface antigen gp200-MR6: single-chain anti-
bodies to evolutionarily conserved determinants disrupt
mouse thymus development. Immunology 96: 236-245.
Ritter, M.A. and Boyd, R.L. (1993) Development in the thymus: it
takes two to tango. Immunol. Today 14: 462-469.
ANTIBODIES TO HUMAN THYMIC EPITHELIUM 93
Ritter, M.A. and Crispe, I.N. (1992) The Thymus. (Oxford, IRL
Press).
Sharif, M., Rook, G., Wilkinson, L.S., Worrall, J.G. and Edwards,
J.C.W. (1990) Terminal N-acetylglucosamine in chronic syno-
vitis. Br. J. Rheumatol. 29:25-31.
Shikhman, A.R., Greenspan, N.S. and Cunningham, M.W. (1993)
A subset of mouse monoclonal antibodies cross-reactive with
cytoskeletal proteins and group A streptococcal M proteins
recognizes N-acetyl-13-D-glucosamine. J. Immunol. 151:
3902-3913.
Shikhman, A.R. and Cunningham, M.W. (1994) Immunological
mimicry between N-acetyl-13-D-glucosamine and cytokeratin
peptides. J. Immunol. 152: 4375-4387.
Shikhman, A.R., Greenspan, N.S. and Cunningham, M.W. (1994)
Cytokeratin peptide SFGSGFGGGY mimics
N-acetyl-l-D-glucosamine in reaction with antibodies and
lectins, and induces in vivo anti-carbohydrate antibody
response. J. Immunol. 153: 5593-5606.
Truneh, A., Albert, E, Golstein, P. and Schmitt-Verhulst, A.-M.
(1985) Early steps of lymphocyte activation bypassed by syn-
ergy between calcium ionophores and phorbol ester. Nature
313:318-320.
Tucek, C.L., Godfrey, D.I. and Boyd, R.L. (1992) Five novel anti-
gens illustrate shared antigenicity between mouse thymic stro-
mal cells, thymocytes and peripheral lymphocytes. Int.
Immunol. 4: 1021-1030.
Underwood, J.R., Pedersen, J.S., Chalmers, P.J. and Toh, B.H.
(1985) Hybrids from normal, germ free, nude and neonatal
mice produce monoclonal autoantibodies to eight different
intracellular structures. Clin. Exp. Immunol. 60: 417-426.
van der Wijngaert, EP., Kendall, M.D., Schuurman, H.-J., Rade-
makers, L.P.H.M. and Kater, L. (1984) Heterogeneity of epi-
thelial cells in the human thymus. An ultrastructural study.
Cell Tissue Res. 237: 227-237.
van Vliet, E., Melis, M. and van Ewijk, W. (1984) Monoclonal anti-
bodies to stromal cell types of the mouse thymus. Eur. J.
Immunol. 14: 524-529.
Volkmann, A., Zal, T. and Stockinger, B. (1997) Antigen-present-
ing cells in the thymus can negatively select MHC class
II-restricted T cells recognizing a circulating self antigen. J.
Immunol. 158: 693-706.

				

